# Dealloying-based interpenetrating-phase nanocomposites matching the elastic behavior of human bone

**DOI:** 10.1038/s41598-017-00048-4

**Published:** 2017-02-02

**Authors:** I. V. Okulov, J. Weissmüller, J. Markmann

**Affiliations:** 1 Institute of Materials Research, Materials Mechanics, Helmholtz-Zentrum Geesthacht, Geesthacht, Germany; 2 Institute of Materials Physics and Technology, Hamburg University of Technology, Hamburg, Germany

## Abstract

The long-term performance of orthopedic implants depends crucially on a close match between the mechanical behavior of bone and of the implant material. Yet, the present man-made materials with the required biocompatibility and strength are substantially stiffer than bone. This mismatch results in stress shielding, which can lead to the loss of bone mass and may even lead to a revision surgery. Here we report a new materials design strategy towards metal-polymer composites that are based on constituents with established biocompatibility and that can be matched to bone. Ti-based nanoporous alloys, prepared by liquid-metal dealloying, are infiltrated with epoxy to form interpenetrating-phase nanocomposites. At up to 260 MPa, their yield strength is technologically interesting for a deformable light-weight material. More importantly, Young’s modulus can be adjusted between 4.4 and 24 GPa, which affords matching to bone. As another parallel to bone, the strength of the composite materials is strain-rate dependent. These findings suggest that the novel composite materials may provide the basis for promising future implant materials.

## Introduction

A commonality of the various engineering materials classes is the trend for the elastic modulus to scale with the strength^1^. For instance, polymers are quite compliant and exhibit relatively low strength, while metals are strong and at the same time quite stiff. The requirement for the ideal orthopedic implant material, among others, includes an “impossible” combination of mechanical properties such as high strength of metals and low stiffness matching that of bone^2–6^. Neglecting the latter, as in the case of commonly applied metallic implants, causes a disproportional distribution of stresses between implant and repaired bone resulting in the stress shielding effect^2,6,7^. Since the health of bone is critically depends on the applied loads, such a scenario may lead to the loss of bone mass and its degradation.

Recently, composites of nanoporous gold and polymer’s where demonstrated as a novel approach towards strong and ductile nanocomposites^8,9^. As it turns out, the material is also unusually compliant, suggesting an unusual deviation from the strength–stiffness scaling. The material’s microstructure features two interpenetrating and geometrically similar phases, a uniform network of nanoscale metallic “ligaments” that are strengthened by their small size, and a contiguous polymer phase. The metal network is formed by dealloying, the selective corrosion of the less noble element from a binary solid solution, and the polymer is infiltrated into the porous metallic preform.

Nanoporous Au and its composites distinguish themselves from fiber-reinforced composites by their excellent deformability, reaching strains up to 0.7 without failure^8,10,11^. While its high cost and mass-density rule out gold as the basis for a structural engineering material, it has recently been shown that micro- or nanoporous structures based on less noble and less dense metals can be fabricated by using a metallic melt as the corrosive medium^12–18^. Here, we combine liquid-metal dealloying (LMD) and polymer infiltration to fabricate moderately strong metal-polymer composites with tunable stiffness matching that of bone. In contrast to existing interpenetrating phase metal-polymer composites based on sintered porous metals^19,20^, the material synthesized by the relatively new LMD method allows considerably larger microstructural tunability of the porous metal scaffolds, in particular ligament size and shape, structure connectivity as well as control of the solid fraction. The microstructural tunability of the dealloyed-based composites affecting the overall mechanical performance features them among existing structural materials. The designed composites are based on Ti, Zr, Ti_50_Zr_50_, Ti_74.4_Nb_25.6_ (at.%) nanoporous metals instead of nanoporous Au. This makes them cost-efficient and light-weight. In the interest of biocompatibility, the components were selected among those currently used in the biomedical field^6,21^. Furthermore, the polymer as well as metal components can be used in many different combinations to ensure even better mechanical and biological compatibility of the composites according to the desired application.

## Results and Discussions

**Figure 1:**
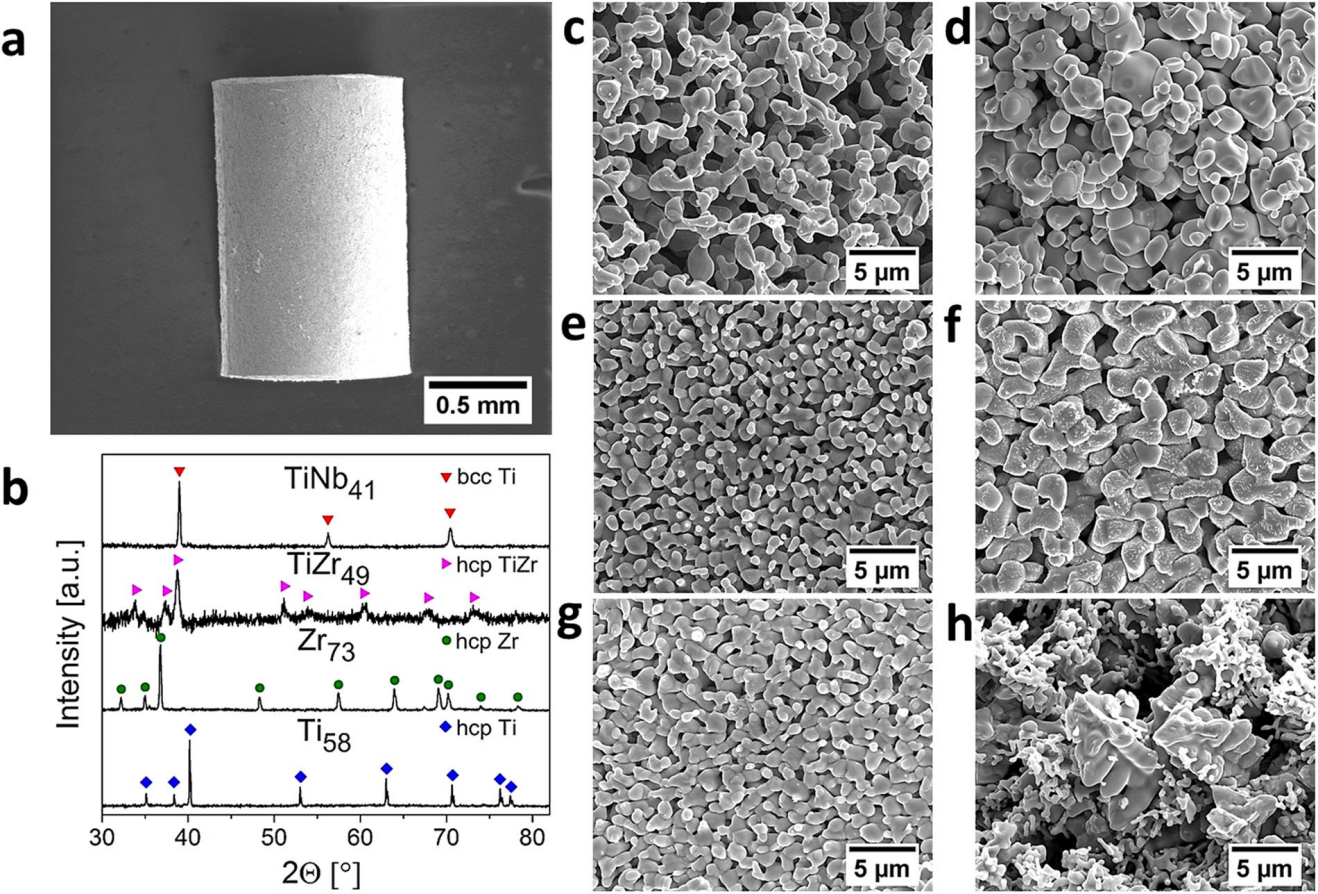
Characterization of porous samples. (**a**) Macroscopic specimen geometry. (**b**) X-ray diffractograms of porous Ti_58_, TiZr_49_, Zr_73_, and TiNb_41_. The diffractograms of Ti_35_ and Ti_62_ are similar to that of Ti_58_ and not shown. Crystallographic phases are marked in the graph. Scanning electron micrographs of Cleavage surfaces of different porous alloys: Ti_35_ (**c**), Zr_73_ (**d**), Ti_58_ (**e**), TiZr_49_ (**f**), Ti_62_ (**g**) and TiNb_41_ (**h**). The number in the sample name reflects its solid fraction.

As detailed in the Materials and Methods section, mm-sized monolythic samples of porous metals with nano- or micrometer- sized pores were made by dealloying pre-shaped master alloy samples in liquid Mg. Figure 1a illustrates the well-defined sample geometry, and the remaining subfigures of Fig. 1 compile representative information on the microstructure. The X-ray diffractograms of Fig. 1b exemplify the single-phase nature of the porous metals. The porous samples fabricated from Ti_20_Cu_80_, Ti_30_Cu_70_, Ti_40_Cu_60_ precursor alloys consist of a single hcp α-Ti phase. The bcc β-Ti phase was stabilized into the porous TiNb samples fabricated from the precursor Ti_22.3_Nb_7.7_Cu_70_ alloy. Dealloying of the Ti_15_Zr_15_Cu_70_ resulted in a formation of porous samples consisting of a solid solution hcp α-Ti(Zr) phase. The porous hcp α-Zr was obtained by dealloying of the Cu_47.5_Zr_47.5_Al_5_ metallic glass. Energy-dispersive analysis of x-ray fluorescence (EDX, results not shown) in a scanning electron microscope (SEM) revealed no residual Cu or Mg in the porous samples.

As can be seen in SEM micrographs such as Fig. 1c–h, the microstructures of the porous metals agree with the uniformly interconnected network structure that has been established of np Au. Characteristic ligament sizes, *L*, are listed in Table 1. The porous TiNb made from Ti_22.3_Nb_7.7_Cu_70_ is an exception, as its microstructure features dendrites that are inherited from the precursor alloy. Since Nb and Cu are immiscible in the solid state^22–25^, microsegregation leads to the Nb-rich dendrites during solidification of the precursor Ti_22.3_Nb_7.7_Cu_70_. Hereafter, the (nominally single-component) porous samples fabricated from the Ti_20_Cu_80_, Ti_30_Cu_70_ and Ti_40_Cu_60_ precursors are referred as Ti_35_, Ti_58_ and Ti_62_, respectively, where the number indicates the metal volume fraction, φ (Table 1). The analogous convention is applied for the porous alloy samples TiZr_49_, Zr_73_ and TiNb_41_, which are made from Ti_15_Zr_15_Cu_70_, Cu_47.5_Zr_47.5_Al_5_ and Ti_22.3_Nb_7.7_Cu_70_, respectively. Samples shrink substantially during dealloying (Table 1), by 0.4 ± 0.2 vol% (for TiNb_41_) up to 35.5 ± 1.7 vol% (for Ti_58_). The amount of shrinkage varies with the Cu content and with the alloying element (Zr or Nb) in the precursor alloy. Lower Cu content enhances the shrinkage while addition of Zr and Nb reduces it. The shrinkage enhances φ and, consequently, the mass-density, ρ, of the porous metals. In all instances, the Ti-based porous alloys reach very low mass densities, between 1.5 and 2.8 g cm^−3^ (Table 1).

**Table 1:** Structural parameters and mechanical properties of the porous metals (without polymer phase). L – ligament size, φ – solid volume fraction, ΔV/V – relative volume shrinkage during dealloying, ρ - mass density, Y – Young’s modulus, σ_Y_ – yield strength.

Porous metals	*L* [μm]	φ [no units]	Δ*V*/*V* [%]	ρ [g cm^−3^]	*Y* [GPa]	σ_Y_ [MPa]
Ti_35_	1.27 ± 0.14	0.35 ± 0.03	24.5 ± 1.3	1.5 ± 0.1	0.3 ± 0.0	10 ± 1
Ti_58_	0.87 ± 0.17	0.58 ± 0.04	35.5 ± 1.7	2.6 ± 0.1	3.0 ± 0.2	65 ± 5
Ti_62_	0.95 ± 0.17	0.62 ± 0.03	19.7 ± 0.9	2.8 ± 0.1	6.0 ± 0.3	72 ± 5
TiZr_49_	1.42 ± 0.14	0.49 ± 0.02	14.7 ± 1.0	2.8 ± 0.1	3.2 ± 0.2	110 ± 10
Zr_73_	1.98 ± 0.42	0.73 ± 0.05	9.6 ± 0.5	4.7 ± 0.1	15 ± 0.9	194 ± 10
TiNb_41_	0.44 ± 0.13	0.41 ± 0.04	0.4 ± 0.2	2.3 ± 0.1	1.0 ± 0.1	22 ± 3

As detailed in Materials and Methods, samples were vacuum-impregnated with two epoxy resins, bisphenol F (BPF) and bisphenol A (BPA). As a small chain-length resin, BPF has low viscosity, facilitating impregnation. BPA is a stronger polymer that is used in commercial fiber-reinforced composites. As has already been shown for np Au, the vacuum impregnation achieves complete filling of the entire pore space with no voids^9^. This was confirmed here by SEM analysis of polished surfaces (not shown) and by analysis of the loading-unloading mechanical tests, discussed below.

The impregnation increases the mass density, ρ. Yet, Table 2 shows that ρ of the titanium-based composites varies about the quite low value of ∼3 g cm^−3^. Remarkably, the densities of the Ti_35_BPA (Ti_35_ infiltrated by BPA) and Ti_35_BPF (Ti_35_ infiltrated by BPF) composites are as low as 2.2 and 2.3 g cm^−3^, respectively; this is substantially less than aluminum alloys (2.69–2.80 g cm^−3^)^26^.

**Table 2:** Mechanical properties and density values of the metal-polymer composites, BPF and BPA epoxies. ρ - mass density, Y – Young’s modulus, σ_Y_ – yield strength and σ_max_ – maximum strength.

Composites and epoxy	ρ [g cm^−3^]	*Y* [GPa]	σ_Y_ [MPa]	σ_max_ [MPa]
Ti_35_BPF	2.3 ± 0.1	4.6 ± 0.3	80 ± 5	135 ± 10
Ti_58_BPF	3.0 ± 0.1	16.9 ± 1.0	160 ± 10	265 ± 20
Ti_62_BPF	3.1 ± 0.1	16.6 ± 1.5	145 ± 10	277 ± 20
TiZr_49_BPF	3.3 ± 0.1	8.2 ± 0.5	263 ± 15	304 ± 20
Zr_73_BPF	5.0 ± 0.1	24.2 ± 0.5	233 ± 15	253 ± 20
TiNb_41_BPF	2.8 ± 0.1	6.3 ± 0.2	95 ± 10	160 ± 10
Ti_35_BPA	2.2 ± 0.1	4.4 ± 0.3	75 ± 5	140 ± 10
Ti_58_BPA	3.1 ± 0.1	15.6 ± 1.0	150 ± 10	260 ± 20
Ti_62_BPA	3.2 ± 0.1	14.6 ± 1.0	218 ± 15	315 ± 20
TiZr_49_BPA	3.4 ± 0.1	10.6 ± 0.5	264 ± 15	347 ± 20
Zr_73_BPA	5.0 ± 0.1	13.8 ± 1.0	189 ± 10	189 ± 10
TiNb_41_BPA	2.9 ± 0.1	7.1 ± 0.3	106 ± 10	191 ± 20
BPF epoxy (10^−4^ s^−1^)	—	1.38 ± 0.2	51 ± 3	51 ± 3
BPF epoxy (10^−1^ s^−1^)	—	1.7 ± 0.3	87 ± 5	87 ± 5
BPA epoxy^38^	—	1.25 ± 0.3	56 ± 3	56 ± 3

**Figure 2:**
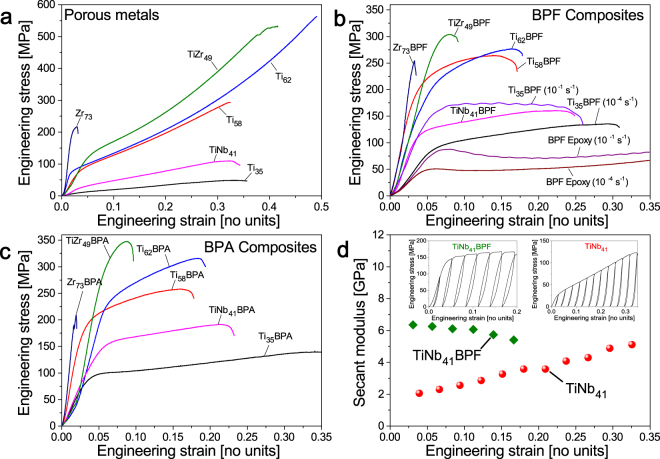
Mechanical behavior of porous metals and composites, probed by compression tests. Strain rates are 10^−4^ s^−1^ unless otherwise indicated. (**a**) Porous metals, compressive stress-strain curves. (**b**) BPF-based composites, compressive stress-strain curve. Note strain-rate sensitivity of strength, as exemplified by the data for Ti_35_BPF. Also shown is data for pure BPF samples (no metal) of similar geometry, illustrating the strain-rate sensitivity of the epoxy. (**c**) BPA-based composites, compressive stress-strain curves. (**d**) Composites TiNb_41_ and TiNb_41_BPF, secant modulus plotted against engineering strain for composite (inset: loading-unloading stress-strain curves for TiNb_41_BPF (left) and TiNb_41_ (right)).

Figure 2a compiles results of mechanical tests on the porous metals. While the porous Zr sample fails in an almost brittle fashion, after a plastic strain of merely ~1%, the compressive stress-strain curves of all other materials are qualitatively similar to those of the model studies on np Au^11,27^. Specifically, all Ti-based porous materials exhibit excellent deformability, with strains of several 10% prior to failure. Consistent with this deformability is the pronounced strain-hardening, which promotes uniform plastic flow. Table 1 compiles the yield strength, σ_Y_, and Young’s modulus, *Y*, of the porous metals. In view of the Gibson-Ashby scaling relations^28^, the data for elemental porous Ti confirms qualitatively the expected enhancement in strength at higher φ. It is also seen that the Ti-based porous alloys tend to be stronger than elemental porous Ti at comparable φ. This is compatible with solid solution hardening in the porous alloys.

The yield strengths of porous TiZr_49_ and Zr_73_ reach 110 MPa and 193 MPa, respectively. These values significantly exceed the strength of commercial macroporous metals, such as foamed aluminum^29^. Furthermore, the yield strength of our composites is about 2 to 4 times higher when compared with these of the previously reported metal-polymer composites possessing Young’s modulus comparable with that of bone^19,20,30^. The high-strength of our materials has a natural explanation in their rather high (compared to macroscopic metal foams) solid fraction, along with the known trend for high-strength at small structure size^31–33^. Similarly to the strengths, Young’s moduli of the porous metals vary widely, from 0.3 GPa for Ti_35_ to 15 GPa for Zr_73_ (Table 1).

We now turn to the mechanical behavior of the composites, as shown in Fig. 2b–d. It is immediately obvious that each composite is several times stronger than its porous metal counterpart (see also Table 2). For example, σ_Y_ of the TiZr_49_BPA composite, 264 MPa, is twofold and σ_Y_ of the Ti_62_BPA composite, 218 MPa, is threefold higher than σ_Y_ of the respective porous metals. A comparison of the trends for strength versus porous metal composition suggests that the composites inherit strength from their metal scaffold. In particular, TiZr_49_ being stronger than TiNb_41_, forms the TiZr_49_BPA composite which is stronger than the TiNb_41_BPA composite. The metal-polymer composites are highly deformable in compression. In particular, the strain to failure is ∼0.35 for Ti_35_BPA and remains as high as 0.2 for Ti_62_BPA, one of the strongest composites.

The distinction between the mechanical behavior of porous metals and composites appears in particular, when one inspects the stiffness data. Figure 2d shows *Y* of the porous metal TiNb_41_ and of the composite TiNb_41_BPF at different plastic strains. *Y* was obtained as a secant modulus in compression tests with interspersed load/unload segments, see insets in the figure. Note first that *Y* of TiNb_41_ increases with increasing strain; similar behavior in np Au has been explained as the result of densification of the porous structure^10,34^. By contrast, *Y* of the TiNb_41_BPF composite even slightly decreases over increasing strain. Note that the closing of voids in case of incomplete infiltration of the porous metal scaffold by polymer would cause *Y* to increase with plastic compression. The observation of decreasing *Y* thus confirms complete infiltration.

Even though they are relatively strong, the composites exhibit low values of Young’s modulus (Table 2). For example, the TiZr_49_BPA and Ti_62_BPA composites have *Y* = 10.6 and 14.6 GPa, respectively. Furthermore, the σ_Y_ of the composites exhibit a notable dependence on the strain rate. This is exemplified for Ti_35_BPF in Fig. 2b. The strain-rate sensitivity of the composites has a natural explanation in the known strain-rate sensitivity of strength and flow stress of polymers; this is also exemplified for the BPF epoxy in Fig. 2b and in Table 2.

As a distinguishing feature, the deformation of the porous metals of this study is accompanied by strain-hardening without the plateau stress typical for commercial macroporous metals^29^. The strain-hardening may be linked to a uniform deformation^27^ – as opposed to the localized crush-bands of metal foam – and to a combination of the densification of the porous scaffold and Taylor hardening^11,35^.

The strength and elastic modulus of the metal-polymer composites exceeds those of each constituent alone, confirming an observation that was already reported for np-Au-polymer composites^9^. Note in particular that the strength of the polymer is much lower than that of the composites (Table 2). The increase in strength when combining the porous metal with the polymer may be understood as the consequence of a change in deformation mode of the metal: rather than densifying by a bending-dominated deformation^35^, as in the porous metal, the ligaments in the composite deform along with an essentially volume-conserving macroscopic strain field.

The design strategy of our new composites picks up what has been demonstrated for composites from nanoporous gold and polymer as model materials. By using a titanium-based porous metal scaffold, we here demonstrate that this strategy can indeed be applied to more application-relevant materials, which are both, of substantially lower cost and of substantially lower weight than the earlier gold-based material. The strength of our new material profits from a combination of strengthening by small structure size, the good network connectivity achieved by dealloying, and solid solution hardening. As a distinguishing feature, these new composites are highly deformable and may thus be amenable to non-cutting shaping, a substantial advantage over fiber reinforced composites. They are also distinguished by their isotropic mechanical behavior, a result of the isotropic microstructure.

As a further benefit of the novel metal-polymer composites, their strength and elastic modulus can be tuned within wide intervals by varying the metal fraction, by alloy design and by the choice of the polymer. In this respect, note that the porous metal preforms are highly deformable and that their stiffness changes upon plastic compression. This might be exploited for tuning the mechanical properties by pre-deformation prior to infiltration.

**Figure 3:**
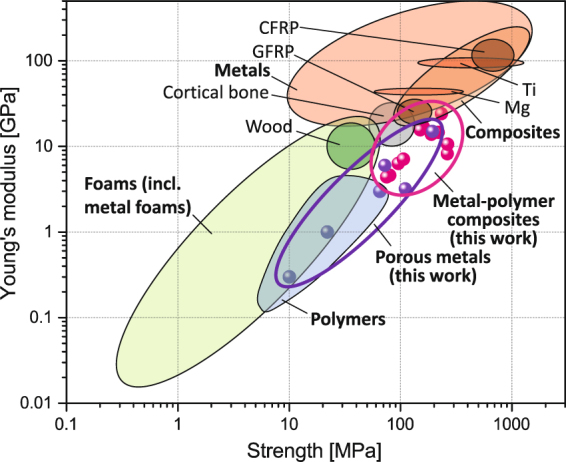
Ashby diagram of Young’s modulus plotted against yield strength demonstrating the unique combination of properties of the metal-polymer composites (Note: CFRP - Carbon-fiber-reinforced polymer, GFRP - glass-fiber reinforced plastic, Mg – magnesium alloys, Ti – titanium alloys).

In the context of biomaterials, the unique properties of the composites are strikingly demonstrated by the Ashby-type diagram plotting stiffness versus strength, Fig. 3. The data from this work covers a substantial area of “white space” at intermediate (rather higher) strength and at rather low stiffness. It is seen that this region bridges a gap between the regions covered by polymers and by metals. The position on the Ashby diagram suggests opportunities for the composites to be used as advanced materials for impact energy absorption, spring or implant application requiring a combination of low stiffness and good yield strength^1,36^.

The stiffness values are particularly remarkable, because they are within the range of stiffness (0.1 GPa–25 GPa) found for bone^3,4^. Since the health and recovery of bone is closely related to the load applied to it, the stiffness of medical implants should ideally match that of the bone to provide a proportional load distribution between the implant and the bone. The low and flexibly tunable elastic moduli of the metal-polymer composites are of high value here because they may enhance the implant lifetime, reducing the risk of revision surgery. Moreover, the notable strain rate sensitivity of strength is another instance where the composites mimic the mechanical behavior of human hard tissues. Likewise to the strength of bones^37^, strength of the composites increases with increasing strain rate. Even more remarkably, the yield strength values of the composites significantly exceed the fracture strength of human bones (*σ*_Y_ = 50–150 MPa)^3,4^ implying a high potential for biomedical application.

Biocompatibility of the metal-polymer composites is determined by their constituents. The metals used are biocompatible and widely applied in orthopedic surgery^2,6^. The BPA resin evokes some concerns regarding its safety, in particular, in the report by the European commission recommending to substitute BPA by BPF as less toxic^21^. However, both resins are used in medicine for fabrication of medical devices and implants. Furthermore, we see no obvious obstacles towards generalizing our materials design scheme to use biocompatible polymers, e.g. polyurethane^9^.

## Conclusions

In summary, we have demonstrated a design strategy of light-weight and cost efficient bicontinuous nano-/microporous metals and alloys by selective corrosion in a liquid metal. Here, we need to emphasize that the tunable bicontinuous microstructure of these nano-/microporous metals is inherited to the fabrication method and, therefore, is unique as well as their corresponding physical properties. Using the fabricated nano-/microporous metals as a base material, we have synthesized metal-polymer composites mimicking the elastic behavior of human bones. As the elastic behavior of bone depends on many factors like, for example, human age, the elastic properties of the composites can be optimized respectively to match the needs. In particular, we have shown that the elastic behavior of the composites can be controlled within a wide range – including that of bone – through optimization of metal fraction, type of polymer as well as type of metal. The perceptible strain rate sensitivity of strength of the composites is another similarity to bone. Metals, as standard orthopedic implant materials, have very low strain rate sensitivity, and our material here profits from the behavior of its polymer component. Overall, the distinguishing combination of properties of our new composites includes intermediate, but technologically interesting yield strengths (75 to 264 MPa), low elastic modulus (4.4 GPa–24.2 GPa), strain rate sensitive strength, good deformability and low density. This strongly suggests opportunities as future advanced implant materials will significantly enhanced performance. However, before application, a comprehensive study of these micro-/nanocomposite materials covering different biomedical, e.g. osteointegration, as well as mechanical, e.g. long-term fatigue, aspects is required.

## Materials and Methods

Precursor alloys were Ti_20_Cu_80_, Ti_30_Cu_70_, Ti_40_Cu_60_, Ti_15_Zr_15_Cu_70_, Cu_47.5_Zr_47.5_Al_5_ and Ti_22.3_Nb_7.7_Cu_70_ (at.%). Rods 1 mm in diameter were prepared from pure metals (99.99%) using arc melting under Ar and a suction casting device (Mini Arc Melter MAM-1, Edmund Bühler, Germany). The Ti_20_Cu_80_ alloy consists of Cu (space group *Fm-3m*) and TiCu_4_ (space group *Pnma*) phases. A metastable TiCu_2_ (space group *Amma*) and TiCu_4_ (space group *Pnma*) phases are found in the Ti_30_Cu_70_ alloy. The Ti_40_Cu_60_ alloy contains the equilibrium Ti_2_Cu_3_ (space group *I4/mmm*) and a metastable TiCu_2_ (space group *Amma*) phases. The as-cast Cu_47.5_Zr_47.5_Al_5_ alloy is amorphous consistently with the literature^39^. The Ti_22.3_Nb_7.7_Cu_70_ alloy consists of β-Ti (space group *Im-3m*), TiCu_4_ (space group *Pnma*) and Cu (space group *Fm-3m*). The Zr_14_Cu_51_ phase (space group *P6*/*m*) is found in the as-cast Ti_15_Zr_15_Cu_70_ alloy.

For dealloying, the as-cast rods were cut to 1.7 mm length by a horizontal diamond wire saw (Model 3032, Well Diamantssäger, Germany) and heated to 1073 K for 300 s, together with plus ~130 mg Mg. For Ti_40_Cu_60_, 1023 K and 360 s were used. An infrared furnace (IRF 10, Behr, Switzerland) and a glassy carbon crucible under Ar flow were used, with heating and cooling rates ~40 K s^−1^. Molten Mg selectively dissolves Cu and Al out of the parent alloys, while Ti, Zr and Nb diffuses along the metal/liquid interface^12,17^. The samples then consisted of hcp Ti, hcp Zr, hcp Ti_50_Zr_50_ or bcc Ti_74.4_Nb_25.6_ and Mg-rich phases dependently on the master alloy. To obtain the porous samples, the Mg phase was removed by etching in 3 M HNO_3_ for 5h.

The composites were prepared by subjecting the porous metal samples to vacuum for 10 minutes and then bringing them in contact with the liquid polymer/hardener mixture, using a vacuum impregnation unit (CitoVac, Struers, Germany). Curing occurred during 20 min at 55 °C followed by at least 48 h at room temperature. Two types of polymers were used, bisphenol F epoxy resin (BER 20, Buehler, Germany, number average molecular weight ≤700 g mol^−1^), mixed 4:1 with amine hardener (BEH 20, Buehler) and bisphenol A epoxy resin (RIMR 135, Hexion Specialty Chemicals, Netherlands, ≤700 g mol^−1^), mixed 10:3 with hardener (RIMH 137, Hexion Specialty Chemicals).

Structural investigation of the precursor alloys and porous samples was performed by X-ray diffraction in Bragg-Brentano geometry (D8 Advance, Bruker, Germany) with Cu-K_α_ radiation and a LynxEye position sensitive detector. Scanning electron microscopy (Nova Nanolab 200, FEI, USA) coupled with energy-dispersive X-ray analysis (EDAX, Germany) explored microstructure and composition. The 1 mm diameter, 1.7 mm long cylindrical samples were tested in compression at room temperature and a strain rate of 10^−4^ s^−1^, using a universal testing device (Z010 TN, Zwick-Roell, Germany). The strain was computed from the relative displacement of the load surfaces, as measured by a laser extensometer (LaserXtens, Zwick). The yield strength of the porous metals and composites was determined at the 0.002 offset strain.
